# Sidestream cigarette smoke and cardiac autonomic regulation

**DOI:** 10.1186/1755-7682-6-11

**Published:** 2013-03-07

**Authors:** Vitor E Valenti, Luiz Carlos M Vanderlei, Celso Ferreira, Fernando L A Fonseca, Fernando R Oliveira, Fernando H Sousa, Luciano M Rodrigues, Carlos B M Monteiro, Fernando Adami, Rubens Wajnsztejn, Luiz Carlos de Abreu

**Affiliations:** 1 Department of Speech Language and Hearing therapy, Faculty of Philosophy and Sciences, UNESP, Av. Higyno Muzzi Filho, 737, Marilia, SP 17.525-900, Brazil; 2 Post-graduation Program in Physiotherapy, Faculty of Sciences and Technology, UNESP, Rua Roberto Simonsen, 305, Presidente Prudente, SP 19060-900, Brazil; 3 Post-graduation Program in Cardiology, UNIFESP, Rua Napoleão de Barros, 715, São Paulo, SP 04024-002, Brazil; 4 Department of Morphology and Physiology, School of Medicine of ABC, Av. Príncipe de Gales, 821, Santo André, SP 09060-650, Brazil; 5 Faculty of Sciences, UNESP, Av. Eng. Luiz Edmundo Carrijo Coube, 14-01, Bauru, SP 17033-360, Brazil

**Keywords:** Autonomic nervous system, Cigarette smoking, Cardiovascular physiology, Air pollution

## Abstract

**Background:**

The literature has already demonstrated that cigarette influences the cardiovascular system. In this study, we performed a literature review in order to investigate the relationship between sidestream cigarette smoke (SSCS) and cardiac autonomic regulation.

**Methods:**

Searches were performed on Medline, SciELO, Lilacs and Cochrane databases using the crossing between the key-words: “cigarette smoking”, “autonomic nervous system”, “air pollution” and “heart rate variability”.

**Results:**

The selected studies indicated that SSCS exposure affects the sympathetic and parasympathetic responses to changes in arterial blood pressure. Moreover, heart rate responses to environmental tobacco smoke are increased in smokers compared to non-smokers. The mechanism involved on this process suggest increased oxidative stress in brainstem areas that regulate the cardiovascular system.

**Conclusion:**

Further studies are necessary to add new elements in the literature to improve new therapies to treat cardiovascular disorders in subjects exposed to sidestream cigarette smoke.

## Background

Environmental tobacco smoke exposure was showed in the literature to be an important contributor to increased cardiovascular morbidity and mortality. The effects of cigarette smoke on respiratory and cardiovascular systems are a relevant matter in the reported several cardiovascular impairment effects [1-3]. Cigarette smoke in the environment is divided into two categories. The sidestream cigarette smoke (SSCS) presents a great number of oxidants and other harmful components, its concentration is much higher than compared to the mainstream smoke. On the other hand, it is known that the mainstream smoke is inhaled by the active smokers and released on the environment while the SSCS is emitted from the cigarette and inhaled by so-called “passive smokers”. Passive smokers are hence exposed to almost the same chemicals in cigarette smoke as active smokers are. Thus, passive smoking increases the risk of cardiac or other related disease in nonsmokers [4].

Most effects of smoking on the autonomic regulation of the heart is attributed to nicotine, an active agent that cigarette triggers acute and chronic cardiovascular responses by through sympathetic activation, resulting in the release of plasmatic catecholamines [5]. As a consequence, there is an increase in heart rate and systemic blood pressure, coronary spasm, increased workload and myocardial oxygen demand with concomitant reduction in this supply. It increases the propensity to arrhythmias and cardiac events in healthy subjects, these changes are attenuated by blocking alpha and beta adrenergic receptors, indicating that these effects are derived from sympathetic activation [6].

Although there are a large number of studies in the literature that investigated the influence of smoking on the autonomic cardiac regulation, it lacks information regarding the effects of SSCS on this mechanism. Thus, in this review we endeavored to gather evidences regarding the effects of sidestream cigarette smoke on cardiac autonomic regulation. We aimed to add elements in the literature in order to better comprehend researchers and to contribute to new therapies.

## Method

### Search strategy and selection

The revisions were made between September 2012 and January 2013. The Medline (via PubMed), Lilacs, Scielo and Cochrane databases were searched using the following subject keywords: “cigarette smoking”, “autonomic nervous system”, “air pollution” and “heart rate variability”. These words were defined by the Medical Subject Headings (MeSH).

The studies were selected by a reviewer and supervised by a senior reviewer. Based on the titles and abstracts, we excluded manuscripts not clearly related to the subject of the review. Thereafter, all the selected titles and abstracts were submitted to a final evaluation, which considered the inclusion criteria, and its reference lists independently checked to identify studies of possible relevance that were not found in the electronic search.

We excluded studies that presented no abstract or full text in English between 2000 and 2012 and literature reviews. As inclusion criteria we considered clinical trials and basic studies that investigated the effects of auditory stimulation on the ANS.

## Results

The electronic search yielded a total of 934 references. Among these references the first elimination resulted in the exclusion of 521 titles and abstracts, which were not clearly related to the subject of review. The titles of the remaining 413 abstracts were submitted to a final evaluation that took into account the inclusion criteria. After the final exclusion of studies that did not investigate the SSCS, we finished the review with five references. The investigation of the reference lists confirmed the absence of relevant documents. Summaries of the main studies analyzed were selected. Table 1 shows the levels of variability and the main results and conclusions of the studies included in this update.

**Table 1:** Main studies regarding the effects of sidestream cigarette smoking on cardiac autonomic regulation

***Authors and year***	***Main conclusions***
Valenti et al., 2010.	Three weeks of exposure to SSCS did not affect baroreflex function in Wistar rats.
Valenti et al., 2010.	SSCS exposure affected the sympathetic and parasympathetic responses to changes in blood pressure in WKY rats while it influenced the sympathetic reaction in SHR.
Cobb et al., 2012.	Waterpipe smoking impairs heart rate variability. Moreover, exposure to smoke components different from nicotine causes the similar effects.
Valenti et al., 2012.	Exposure to SSCS impairs cardiovascular responses through its influence on catalase mechanism into the 4th V.
Ordoñana et al., 2012.	Cardiovascular reaction to environmental tobacco smoke is associated with individual craving in smokers. Psychophysiological responses to environmental tobacco smoke are increased in smokers.

WKY: Wistar Kyoto rats; SHR: Spontaneously Hypertensive Rats; 4th V: Fourth Cerebral Ventricle.

## Discussion

Considering the small number of studies in the literature that investigated the association between SSCS and cardiac autonomic regulation, we endeavored to review recent studies from our group and others groups regarding this issue. The analysis of manuscripts selected for this review showed that exposure to SSCS impairs the autonomic regulation of the heart through the central nervous system and through the periphery.

A first study from our group mentioned in this review failed to report changes in baroreflex function in Wistar rats exposed to SSCS during three weeks, five days per week for 180 minutes per day. The baroreflex sensitivity was compared between rats exposed to SSCS and rats exposed to fresh air [7]. Nevertheless, previous studies indicated that active smoking influences cardiovascular reflexes. It was indicated that cigarette smoking in active smokers increases sympathetic nerve activity through an effect mediated by the central nervous system and also through a direct peripheral mechanism [6]. In the study published by our group, Valenti et al. [7], there was no difference between rats exposed to SSCS and rats exposed to fresh air regarding tachycardic and bradycardic peak in response to decrease and increase in arterial blood pressure, respectively. Furthermore, it was not found difference in sympathetic and parasympathetic baroreflex gain. It is interesting to raise the hypothesis that the period of exposure to SSCS used in our published study (three weeks) was not enough to induce changes in baroreflex sensitivity in Wistar rats. Conversely, a previous investigation that used a similar protocol to expose rats to mainstream cigarette smoke reported that chronic exposure induces cardiac remodeling that is characterized by a decrease in ventricular functional capacity [8]. Taken together, based on the studies cited above, we may propose that cigarette smoke exposure during three weeks is able to damage cardiac function without influence the autonomic regulation of the heart.

Another investigation cited in the review published by our group evaluated the effects of SSCS exposure on sympathetic and parasympathetic responses induced by decrease and increase in arterial blood pressure in Wistar Kyoto (WKY) and spontaneously hypertensive rats (SHR) [9]. At this time, it was found that SSCS exposure over three weeks attenuated the sympathetic and parasympathetic responses in WKY whereas it attenuated the sympathetic responses in SHR. The exposure protocol used in this study indicated that SSCS reduced tachycardic peak, bradycardic reflex and heart rate range in WKY rats, while it influenced the heart rate range and tachycardic peak in SHR. These findings support the suggestion that a period of cigarette exposure lower than 30 days is enough to induce changes in the sympathetic nervous activity on the heart with no alterations on baseline arterial blood pressure. The protocol applied in rats to activate parasympathetic and sympathetic responses induced by changes in arterial blood pressure is well accepted in the literature [9,10]. The method is based on intravenous phenylephrine that enhances arterial pressure and activates the parasympathetic nervous system, leading to the bradycardic reflex and then the bradycardic baroreflex gain is calculated. The intravenous sodium nitroprusside decreases arterial blood pressure and activates the sympathetic nervous system, leading to the tachycardic reflex [11-13]. The difference between the results from this cited study and the other reported in Wistar rats regarding autonomic cardiac responses to SSCS is possible due to the difference between WKY and Wistar rats, since WKY rats come from normotensive offspring of SHR that the NIH investigators acquired from a colony in Kyoto [14].

According to one of the selected manuscripts in this review, Ordoñana and coworkers [15] indicated that exposure to environmental tobacco smoke caused a stable intensification in skin conductance levels and heart rate responses. Furthermore, the authors reported that this intensification was consistently increased in smokers than non-smokers. According to the authors, smokers subjects presented an increase in heart rate that continued regularly higher than their basal levels. The distinction between smokers and no-smokers subjects was more apparent at the end of the exposure to environmental tobacco smoke when the smoke density was the highest, and non-smokers did not present an appropriate response to this stimulation. Previous studies regarding cue-reactivity reported that heart rate enhanced when smokers were exposed to drug-related vs. drug-neutral stimulation [16,17]. It is suggested that this reaction is dependent on the degree of nicotine dependence or the deprivation level [18]. Additionally, Ordonãna and coworkers [15] reported that the increase in heart rate in response to environmental tobacco smoke positively correlated with subjective craving.

In a very elegant and well conducted study, Cobb and colleagues [19] published a cross-over study design with 32 participants that investigated the effects of toxicant exposure to waterpipe smoking on heart rate variability. Heart rate variability is a well accepted method that provides information regarding RR interval, i.e. the interval time between each heart beat [20-23]. Cobb et al. [11] reported that waterpipe smoking acutely impaired heart rate variability, they also concluded that exposure to smoke constituents other than nicotine causes the same malefic effects on the cardiac autonomic regulation. The results cited above support previous studies that evaluated the acute effects of smoking a single cigarette [24-26]. It was observed an increase in the low frequency/high frequency ratio after smoking a single cigarette, the baseline levels of this index recovered around 20 minutes after smoking cessation.

In order to investigate the physiological mechanisms involved in SSCS induced cardiovascular responses our group tried to evaluate the effects of catalase inhibition into the fourth cerebral ventricle on cardiovascular responses in rats exposed to SSCS [27]. It was reported that central catalase inhibition increased heart rate during the first 5 minutes in rats exposed to fresh air while it increased heart rate and attenuated bradycardic peak during the first 15 minutes in rats exposed to SSCS. As a main conclusion, it was suggested that SSCS exposure increased the cardiovascular responses caused by catalase inhibition into the fourth cerebral ventricle, proposing a central mechanism that involve oxidative stress. In this context, a previous study reported that exogenous H_2_O_2_ into the fourth cerebral ventricle influenced heart rate and arterial blood pressure [28]. The results observed in this study support the hypothesis of the nose anatomy, which is based on the transport of exogenous agents into the brain [29]. The dendritic buttons and protuberant cilia of the olfactory bulb make contact with its surface area. The olfactory cells are first-order neurons that send axons to the brain without intervening synapse. However, it was not possible to confirm which brainstem area was affected by SSCS exposure. According to the anatomy of the fourth cerebral ventricle, a drug injected in this are present a preference for the parasympathetic activity on the heart rate [30]. Future investigations are necessary to confirm this hypothesis.

Another important issue to be addressed is the relationship between SSC smoke and endothelial function in the cardiac autonomic regulation. Distinct types of regulation are suggested such as autonomic and hormonal modulation. The variation of regulatory processes implies in interactions [31]. In this context, nitric oxide is produced in the endothelium and in the heart, it is indicated that the triggering type is involved in the role of this substance in the cardiovascular system, affecting both the heart and the endothelium [32]. Also, nitric oxide production is influenced by the expression of calcium/calmodulin-dependent kinase IV, which regulates blood pressure [33]. Masahiko et al. [34] failed to find a significant effect of passive smoke exposure on the forearm vascular responses induced by vasodilators in non-smokers subjects. On the other hand, Argacha and coworkers [35] reported that tobacco SSCS extract impaired endothelium-dependent relaxation in the isolated aorta of rats. The authors observed that it was related to increased oxidative stress and to tobacco and the increased superoxide production was not accompanied with changes in acetylcholine-induced relaxation. The genetic polymorphism is an important mechanism that is worth to be referred. Gairola and colleagues [36] investigated the effects of SSCS exposure on atherosclerotic lesions in apoE−/− mice. The authors reported an elevation of atherosclerotic injury progress induced by SSCS in this strain, suggesting that a genetic component is also involved. Taken together, it is suggested that the interaction between autonomic and endocrine systems may be involved in the induced-SSCS cardiovascular injuries.

Our study presents some points that are worth to be pointed. As a study review, it does not add new elements in the literature. Nonetheless, it raises important hypothesis for further investigation regarding this issue. Although great part of the selected studies was performed in animals, the mechanisms observed are currently supported, however, it needs additional clarification for improvement in clinical application. We decided to not select all studies linking cigarette and cardiovascular system in order to focus our objective that was to investigate the relationship between SSCS and autonomic regulation of the heart.

## Conclusion

This review showed that SSCS exposure produces changes on autonomic nervous system components that regulate the heart rate, indicating that there is a reduction of a parasympathetic system activity. Nevertheless, the results about the sympathetic activity are inconclusive. Further studies are necessary to clarify this mechanism.

## Competing interests

The authors declare that they have no competing interests.

## Authors’ contributions

All authors participated in the acquisition of data and revision of the manuscript. All authors determined the design, interpreted the data and drafted the manuscript. All authors read and gave final approval for the version submitted for publication.
